# Heat Inactivation of Listeria monocytogenes on Pecans, Macadamia Nuts, and Sunflower Seeds

**DOI:** 10.1128/Spectrum.01134-21

**Published:** 2021-10-13

**Authors:** Meghan Den Bakker, Henk C. den Bakker, Francisco Diez-Gonzalez

**Affiliations:** a Center for Food Safety, University of Georgia, Griffin, Georgia, USA; University of Minnesota

**Keywords:** thermal inactivation, *Listeria monocytogenes*, tree nuts, *D* value, roasting

## Abstract

This project was undertaken to determine the kinetic parameters of thermal inactivation of Listeria monocytogenes on pecans, macadamia nuts, and sunflower seeds subjected to heat treatments simulating industry processes. Five strains were grown in nonselective medium, mixed, and resuspended before inoculating macadamia nuts, pecans, and sunflower seeds (6 to 9 Log CFU/g). Redried inoculated pecans and macadamia nuts were heated in an oven at a temperature range of 90 to 140°C. Unshelled sunflower seeds were heated in sunflower seed oil. The thermal inactivation was determined by measuring viable cell counts using standard microbiological methods. Average count data were fit to the log-linear model, and thermal-death kinetics were calculated. On pecans, the viable *Listeria* counts were reduced by 3 and 3.5 Log CFU/g after 40 min at 110°C and 8 min at 140°C, respectively. On macadamia nuts, the L. monocytogenes population was reduced by 5 Log CFU/g after 20 min at 120°C. Unshelled sunflower seeds were subjected to heat treatment via a hot-oil bath. On sunflower seeds, >7 Log CFU/g reductions were observed after 15 min at 120°C. The thermal resistance (*D* value) for inactivation on pecans at 140°C was 3.1 min and on macadamia nuts at 120°C was 4.4 min. The inactivation of L. monocytogenes was influenced by the kind of nut or seed. These results suggest that L. monocytogenes has a relatively high thermal tolerance. The findings from this study will contribute to the assessment of the effectiveness of heat treatment for control of this pathogen on nuts and seeds.

**IMPORTANCE** Listeria monocytogenes is a major concern for the food industry in ready-to-eat (RTE) foods. In recent years, large-scale recalls have occurred with contaminated sunflower seeds and macadamia nuts that triggered product withdrawals. These events stress the importance of understanding *Listeria’*s ability to survive heat treatments in these low-water activity foods. Nuts and seeds are subjected to a variety of thermal treatments typically referred as roasting. To date, no listeriosis outbreak has been linked to nuts and seeds, but the recent recognition that this pathogen can be detected in commercial products stresses the need for research on thermal treatments. The characterization of heat inactivation kinetics at temperatures typically used during roasting processes will be very beneficial for validation studies. This manuscript reports inactivation rates of L. monocytogenes strains inoculated onto macadamia nuts, sunflower seeds, and pecan halves subjected to temperatures between 90 and 140°C.

## INTRODUCTION

Listeria monocytogenes is a major concern for the food industry in ready-to-eat (RTE) foods. This pathogen is widely distributed in nature, has a unique ability to inhabit multiple niches, and often contaminates food products in some of the last steps before consumption. The majority of listeriosis foodborne outbreaks have been linked to deli meats and dairy food products (1), but in recent years, listeriosis outbreaks have been associated with diverse RTE foods. L. monocytogenes infection cases have not been associated with low-water activity (a_w_) foods, but it is recognized that as RTE foods, they may be susceptible to contamination and may be used as ingredients for other high-moisture foods.

In the last 20 years, an increased number of foodborne outbreaks linked to dry or low-water activity foods have been reported. Among this category of foods, tree nuts and seeds have sporadically been associated with Salmonella outbreaks (2, 3). Because of the continued outbreak incidence of salmonellosis linked to some dry foods, extensive research has been conducted on peanuts, almonds, pistachios, and pine nuts (4–6). Salmonella’s inactivation during storage and heating has been thoroughly characterized in several of those tree nuts and peanuts (7). Those reports have clearly demonstrated that Salmonella has a unique ability to survive for a very long time and at relatively high temperatures at low water activity.

The increased outbreak incidence has led the food industry to expand the frequency of testing of low-water activity foods, which has resulted in a greater number of product recalls. Since many of those products include RTE nuts and seeds, routine food testing has involved not only Salmonella, but also L. monocytogenes (8). In 2016, a large-scale recall of multiple products was due to detection of L. monocytogenes in sunflower seeds (SS) that were commercialized individually and as an ingredient of different food products, which included granola bars, salads, snack mixes, nut spreads, and butters (9). In addition to that massive recall, other small recalls have been linked to nut butters and to sunflower seeds. Similarly, macadamia nut (MN) contamination in 2017 triggered another large food recall involving several product types (10). Despite the fact that there has never been a documented case of listeriosis linked to a seed or nut product, the detection of L. monocytogenes in commercial lots demands a better understanding of its prevalence, survival, and thermal tolerance in these commodities.

Additional product recalls have been linked to other nuts and seeds, such as contaminated cashew nuts and nut butters (11, 12), but the prevalence of *Listeria* in RTE nuts is largely unidentified. *Listeria* is known to be able to survive in the environment for a long time, and recent reports observed prolonged viability on dry foods during storage. The count of inoculated L. monocytogenes bacteria on pecans and peanuts remained relatively unchanged at −24 and 4°C for a year, but at 22°C, its viability declined gradually from 4 Log CFU/g to less than 1 Log CFU/g after 150 days of storage (10). Salazar et al. (13) studied the survival of L. monocytogenes in pine nuts and sesame seeds and reported viable counts of more than 4 Log CFU/g (initial inoculum of 9 Log CFU/g) after 180 days of storage at different relative humidities. A recent study corroborated the prolonged survival of L. monocytogenes in pistachios and reported approximately 2 Log CFU/g from an initial 6 Log CFU/g inoculum after 195 days at 23°C and 5 Log CFU/g after 336 days at 4°C (14). These findings clearly support the idea that L. monocytogenes can remain viable in nuts and seeds during storage.

The effect of heating on the viability of pathogenic bacteria on low-water activity foods has been studied extensively for Salmonella, but relatively few studies have been conducted to elucidate the thermal tolerance of L. monocytogenes. Kenney and Beuchat determined the values for thermal resistance (*D* values) of L. monocytogenes subjected to 60°C for 26 and 37 min when inoculated into peanut butter and chocolate/peanut spread, respectively (15). These values were 5- to 6-fold smaller in high-moisture products, such as milk and chocolate milk. The heat inactivation kinetics of a mixture of 3 L. monocytogenes strains were determined in an almond meal model at temperatures that ranged from 75 to 90°C (16). In the latter study, *D* values of 13.2 min were measured at 90°C and at a constant a_w_ of 0.25. The lack of data on the thermal tolerance of *Listeria* in dry foods clearly stresses the importance of conducting studies aimed at determining inactivation kinetics.

Nuts and seeds are often subjected to a thermal treatment, such as roasting and oil immersion. Pecans, macadamia nuts (MN), and sunflower seeds (SS) are often heated with dry air at 100°C or higher temperatures. It is critical to determine the potential impact that roasting steps can have in reducing L. monocytogenes. To our knowledge, there has never been a documented listeriosis case due to consumption of pecans, macadamia nuts, or sunflower seeds, but because of the reported occurrences of recalls, further investigations are clearly warranted. The present study was undertaken to characterize the kinetic parameters of thermal inactivation of L. monocytogenes on pecans, MN, and SS.

## RESULTS

**FIG 1:**
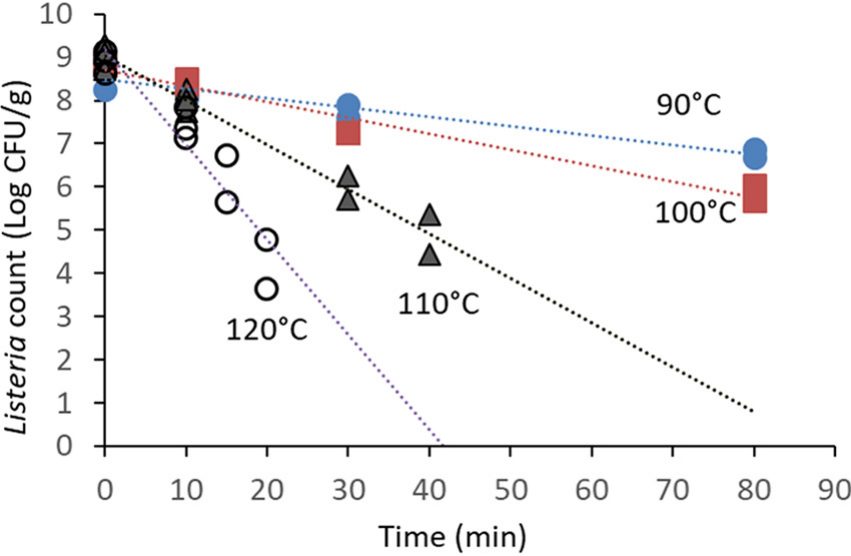
Thermal inactivation of L. monocytogenes on macadamia nuts (MN) in a dry-air oven. Whole MN were surface inoculated with a mixture of five strains, allowed to dry for 24 h, and stored at room temperature for 4 to 6 days before thermal treatment.

**FIG 2:**
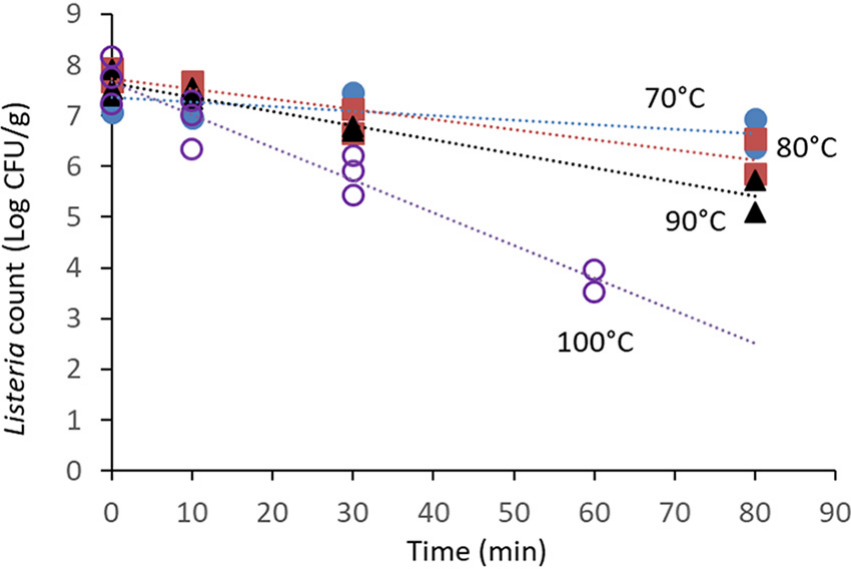
Thermal inactivation of L. monocytogenes on in-shell sunflower seeds (SS) in a dry-air oven. In-shell sunflower seeds were surface inoculated with a mixture of five strains, allowed to dry for 24 h, and stored at room temperature for 4 to 6 days before thermal treatment.

**FIG 3:**
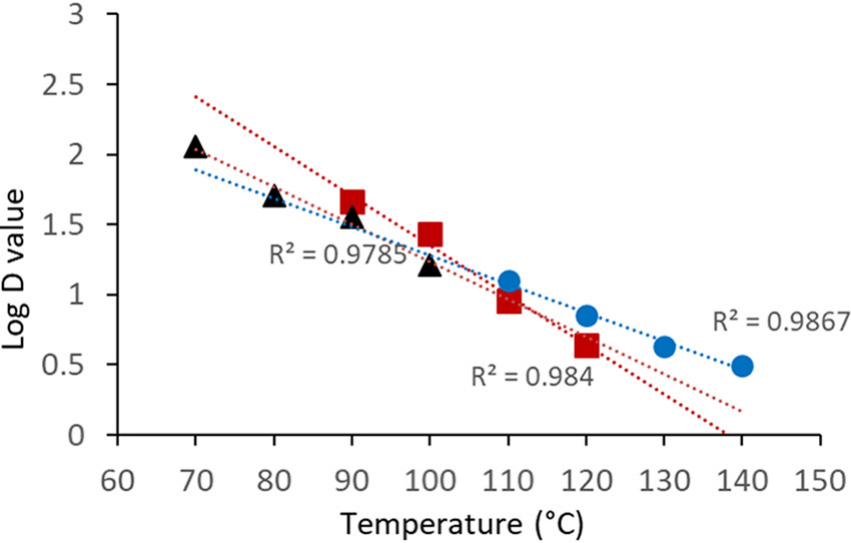
Relationships between temperatures and log *D* values for the calculation of *Z* values of the thermal inactivation of L. monocytogenes on pecan halves (circles), macadamia nuts (squares), and in-shell sunflower seeds.

The initial water activities (a_w_s) of pecans, MN, and SS were 0.62, 0.44, and 0.53, respectively (Table 1). After inoculation and redrying, their water activities increased by 0.04, 0.13, and 0.04, respectively. The water activities at the end of thermal treatments declined to less than half of the original measurements in pecans and MN, but in the case of SS, the final water activity was reduced to 0.06. The count of L. monocytogenes on MN subjected to a temperature of 90°C was reduced by almost 2 Log CFU/g after 80 min (Fig. 1), but it declined by 4.5 Log CFU/g within only 20 min at 120°C. Thermal inactivation on MN could be modeled by using the log-linear model with *R*^2^ values greater than or equal to 0.90 (Table 1). *D* values ranged from 4.5 min at 120°C to 46.2 min at 90°C (Table 2). Thermal inactivation of *Listeria* on in-shell SS also fit the log-linear regression model, with consistent *R*^2^ values of 0.95 or greater. A different temperature range was used with in-shell SS because of current industry practices, which use temperatures from 70 to 100°C. The corresponding *D* values were 113.6 and 13.5 min at 70 and 100°C, respectively, and approximately 4-Log CFU/g reductions were observed after 60 min at 100°C (Fig. 2). The *Z* values (the increase in temperature needed to reduce *D* value by 90%) of *Listeria* inactivation in MN and in-shell SS were 28.40 and 35.46°C, and the *R*^2^ values of their linear regressions were 0.984 and 0.978, respectively (Fig. 3).

**TABLE 1:** Water activity measurements of pecan halves, macadamia nuts, and in-shell sunflower seeds during three stages of experimentation

Sample type	w_a_ (mean ± SD) at indicated stage^*a*^		
	Preinoculation	Postdrying/preheating	Postheating
Pecans	0.62 ± 0.05	0.66 ± 0.02	0.30 ± 0.09
Macadamia nuts	0.44 ± 0.02	0.58 ± 0.04	0.40 ± 0.02
Sunflower seeds	0.53 ± 0.06	0.58 ± 0.04	0.06 ± 0.02

a All measurements were taken on ground samples equilibrated to 21 to 24°C.

**TABLE 2:** Rates of Listeria monocytogenes inactivation on seeds and nuts heated in a dry oven

Temp (°C)	Value for^*a*^:					
	Pecan halves		Macadamia nuts		Sunflower seeds	
	*D* value (95% CI) (min)	*R* ^2^	*D* value (95% CI) (min)	*R* ^2^	*D* value (95% CI) (min)	*R* ^2^
70	ND	ND	ND	ND	113.6 (52.9–716)	0.99
80	ND	ND	ND	ND	50.3 (35.2–87.1)	0.95
90	ND	ND	45.9 (35.0–66.2)	0.99	35.7 (29.8–44.5)	0.97
100	ND	ND	27.0 (22.5–33.5)	0.95	14.5 (13.0–19.3)	0.99
110	12.5 (11.7–17.1)	0.99	9.4 (8.4–11.4)	0.93	ND	ND
120	7.1 (5.5–9.9)	0.99	4.4 (3.5–6.4)	0.90	ND	ND
130	4.3 (3.3–4.8)	0.98	ND	ND	ND	ND
140	3.1 (2.8–3.7)	0.97	ND	ND	ND	ND

a The rates of inactivation were calculated using a log-linear model. CI, confidence interval; ND, not determined.

**FIG 4:**
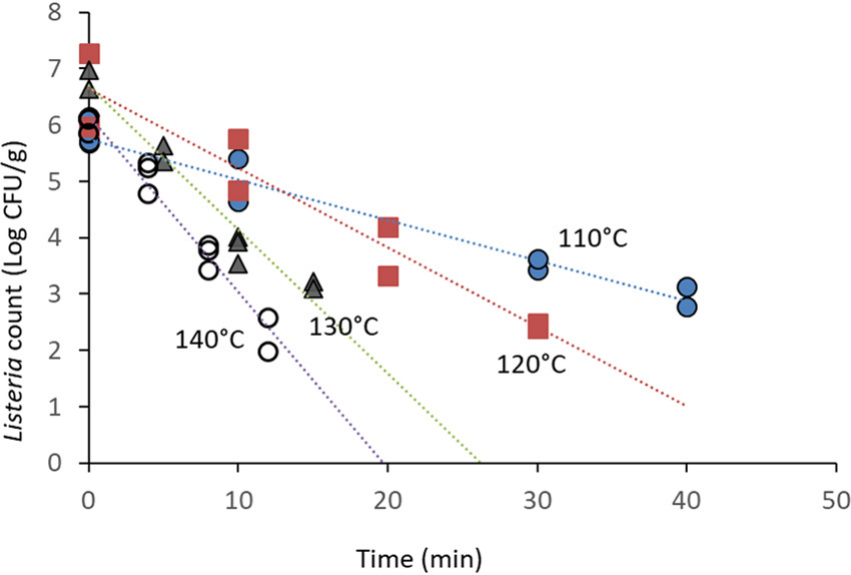
Thermal inactivation of L. monocytogenes on pecan halves in a dry-air oven. Pecan halves were surface inoculated with a mixture of five strains, allowed to dry for 24 h, and stored at room temperature for 4 to 6 days before thermal treatment.

The initial counts on MN and in-shell SS were not markedly affected by the bacterial cultivation method (liquid or agar), and relatively large populations (approximately 8 CFU/g) were stable for several days after inoculation. Five days postinoculation, in-shell SS had average counts of 8.7 CFU/g when inoculated from agar-grown cultures; in comparison, the count on SS inoculated from liquid cultures was 7.9 CFU/g. However, when pecan halves were inoculated with *Listeria* cultures grown in liquid medium, the initial count after drying was reduced from an estimated 8 Log CFU/g to less than 3 Log CFU/g (data not shown). The counts on pecan samples inoculated with cell suspensions collected from the surface of tryptic soy agar (TSA) plates and then dried were only reduced to 6 to 7 Log CFU/g. This is why the thermal inactivation with pecans in this report was conducted using cultures grown in solid medium and why the initial inoculum counts in pecans were lower than those in the MN and SS experiments. At 110°C, the lowest temperature tested in pecans, the L. monocytogenes counts were reduced by 3 Log CFU/g after 40 min of heat treatment (Fig. 4). Similar viability reductions were measured after only 15 min at 130°C. The *D* values of *Listeria* inactivation on pecan halves ranged from 3.1 min at 140°C to 12.5 min at 110°C, and the *Z* value in this range of temperatures was 48.50°C (*R*^2^ = 0.987).

**FIG 5:**
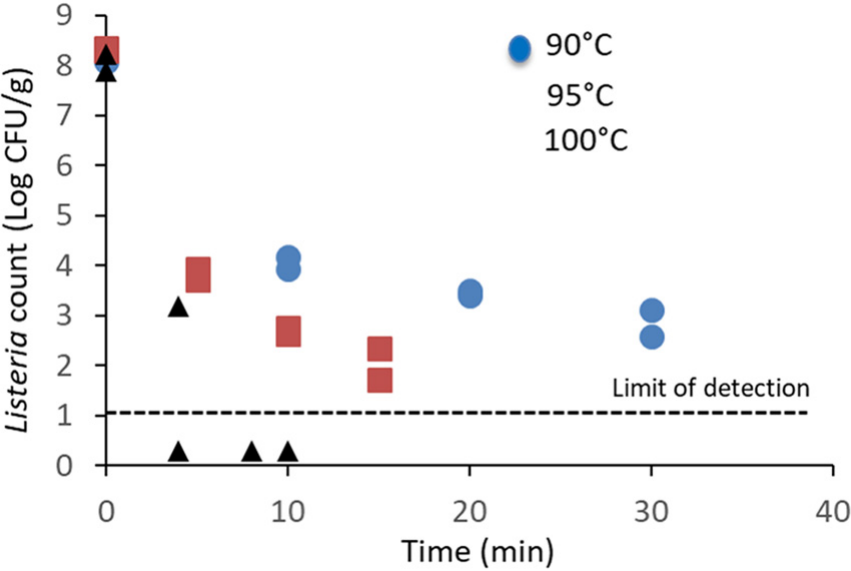
Inactivation of L. monocytogenes on unshelled sunflower seeds (SS) heated in SS oil. Unshelled sunflower seeds were surface inoculated with a mixture of five strains, allowed to dry for 24 h, and stored at room temperature for 4 to 6 days before thermal treatment.

Unshelled SS were the only food matrix heated in oil because of current industry practices. The viable counts of the *Listeria* cocktail inoculated onto SS were rapidly inactivated by more than 5 Log CFU/g at 90°C after 30 min (Fig. 5). If the temperature was increased to 100°C, no measurable counts were determined after heating for only 8 min. Thermal-kinetic parameters could not be determined due to lack of linearity and a low regression coefficient with the Weibull model.

## DISCUSSION

According to the U.S. Department of Agriculture’s Economic Research Service, the consumption of the most popular tree nuts by the American consumer averages 1.67 kg per person every year. The most popular nut varieties are almonds, walnuts, pecans, pistachios, and macadamias, with *per capita* consumption amounts of 0.63, 0.19, 0.15, 0.15, and 0.03 kg, respectively, in 2016 (17). Since 1970, the consumption of tree nuts has increased because of their nutritional value, and in recent years, the almond consumption rate in particular increased rapidly because of the demand for almond-based milk-alternative beverages. The *per capita* consumption of pecans has remained relatively steady for over 50 years, and the total in-shell pecan production in 2018 was 136,000 metric tons (18).

Because of the economic and nutritional importance of nuts and seeds, it is critical to continue advancing our understanding of microbial food risks. To date, there is no documented case of gastrointestinal infection caused by pecans, MN, or SS, but all three of these commodities have been involved in product recalls due to detection of Salmonella or *Listeria* (9, 19, 20). Pecans have frequently been targeted for prevalence, survival, and thermal-inactivation research studies. Only a few foodborne-pathogen-related studies involving MN and SS have been reported. The majority of published works using pecans, MN, and SS have targeted Salmonella.

The prevalence of Listeria monocytogenes in nuts and seeds is largely unknown, and there are only two studies that have reported large-scale testing of commercial products for the presence of Salmonella. A survey conducted in the United Kingdom in 2007 to 2008 found only one sample of SS positive for Salmonella and four E. coli-positive samples from 976 retail SS samples (21). In another survey conducted in the United States in 2014 to 2015, researchers tested 597 pecan and 338 MN samples, and they found 0 and 15 Salmonella-positive samples, respectively (22). Because of the recent seed and nut recalls, it is critical to conduct survey assessments to determine the prevalence of pathogens in their supply chains.

The thermal tolerance and inactivation kinetics of Listeria monocytogenes have been studied extensively under conditions of high moisture content and in high-water activity foods (23, 24). Because of *Listeria*’s association with dairy food outbreaks, the effectiveness of pasteurization on inactivation has been thoroughly documented. Using linear modeling, *D* values of 1.5 s were estimated at 72.7°C, which validated a pasteurization time of 15 s (24). Under high-moisture conditions, the typical *D* values of L. monocytogenes’ thermal inactivation reported in the literature have been always less than 1 min at 65°C (23, 25). The lack of listeriosis cases linked to low-water activity foods explains the very limited number of thermal-inactivation reports currently available.

The thermal tolerance of Salmonella has been extensively characterized in multiple food matrices and conditions. Santillana Farakos et al. (26) compiled and analyzed a large number of publications in a review paper on Salmonella’s thermal inactivation. From this paper and many other publications, it is well established that Salmonella’s thermal tolerance is dramatically enhanced under lower-water activity conditions, and kinetic inactivation parameters, such as *D* value and δ value (time needed to reduce the initial 1 log reduction in viable count), can be several orders of magnitude larger than those obtained under high-moisture conditions. For example, Salmonella cells equilibrated on glass beads and heated at 75°C at a water activity of 1.0 were inactivated at a δ value of 29 s, while a δ value of 51 min was obtained with cells subjected to a water activity of 0.11 (27). In addition to Salmonella, the thermal inactivation of other bacteria at low water activity has yet to be elucidated.

There are no more than 10 publications reporting the thermal-inactivation kinetics of L. monocytogenes under low-water activity conditions. Kenney and Beuchat (15) reported for the first time that the thermal tolerance of L. monocytogenes increased under low-water activity conditions. At 60°C, they determined a thermal-inactivation *D* value of 3.2 min in a high-moisture peanut beverage and a *D* value of 26 min in peanut butter with a water activity of 0.32. In another study, *D* values of L. monocytogenes in four dry foods (confectionery, culinary, chicken meat powder, and pet food) at 80°C ranged from 0.6 to 2.31 min (28). The authors compared the *D* values of Salmonella, which were 3- to 5-fold greater than *Listeria*’s in confectionary and chicken meat powder. Ballom et al. (29) determined L. monocytogenes’ *D* values at 80°C in nonfat dry milk to be 4.3 and 14.6 min at a_w_s of 0.45 and 0.25, respectively. In the most recent report on thermal inactivation of L. monocytogenes, Zhu et al. (16) measured *D* values in almond meal at a_w_s of 0.25 of 60, 28, and 13 min at 80, 85, and 90°C, respectively, under experimental conditions that kept the a_w_ constant. Comparing the different findings described above, multiple factors, such as the food matrix, experimental conditions, and water activity, seem to influence thermal-kinetic parameters.

Our study aimed to determine the thermal inactivation of L. monocytogenes bacteria inoculated onto the surface of pecan nut meats, macadamia nut meats, and sunflower seeds at temperatures that resembled industrial roasting. In all three cases, the relatively high *R*^2^ values indicated that the viability reduction followed a log-linear pattern, and we were able to calculate *D* values. We corroborated the observations of the reports described in the previous paragraph that L. monocytogenes’ thermal tolerance was markedly enhanced under low-water activity conditions. In whole nuts and seeds, extremely high survival rates were observed even at temperatures above 400°C. On MN and SS, the *D* values at 90°C were 3- and 4-fold greater than the largest value observed in almond meal by Zhu et al. (16). These differences were probably due to the use of whole nuts with surface inoculation in contrast to ground almonds with uniform distribution of the inoculum.

The faster inactivation of *Listeria* in SS heated in oil clearly suggested that the heating medium has a great impact on thermal tolerance. When in-shell SS were treated with hot air at 100°C, 4-Log CFU/g reductions were observed after 60 min, but when heated by oil, the viable count was reduced by 6 Log CFU/g after only 10 min. There is almost no other study that has compared the inactivation of L. monocytogenes by air and oil heating, but other researchers have reported differences in Salmonella inactivation between these two heating media. Beuchat and Mann (30) determined that oil heating for 1.5 min at 127°C resulted in greater than 5-Log CFU/g reductions on pecan halves inoculated with Salmonella, but similar reductions were only observed with hot air at 160°C. Another study reported a similar effectiveness of oil heating in almonds, where heating at 121°C for 2 min also resulted in 5 Log CFU/g of inactivation (31).

In the only published study that has investigated thermal inactivation of L. monocytogenes on macadamia nuts, vacuum steam pasteurization was used (32). That study reported reductions in the *Listeria* viable counts of 4.8 and 5.2 Log CFU/g on MN after treatments at 72°C for 38 min and at 82°C for 12 min, respectively. Comparing the thermal susceptibilities based on our results with those in the latter publication, it can be hypothesized that steam pasteurization is much more effective in killing L. monocytogenes on MN.

In summary, L. monocytogenes’ thermal tolerance on low-water activity foods like macadamia nuts, sunflower seeds, and pecans was relatively high when hot air was the treatment medium. According to our findings, *Listeria* may be capable of surviving roasting temperatures below 100°C and perhaps even higher, depending on the treatment time. This report provides some kinetic data that would be useful for the development of validation studies to assess the effectiveness of industrial roasting processes for macadamia nuts, sunflower seeds, and pecans. Further work is needed to evaluate the influence of other variables, such as a type of roasting that involves a dynamic process and forced hot air. These conditions would more closely simulate industrial roasting.

## MATERIALS AND METHODS

### Bacterial strains and growth conditions.

Five Listeria monocytogenes strains were obtained from the culture collection at the Center for Food Safety, University of Georgia, and used in this study. They included strain G1091, isolated from coleslaw, strain Scott A, strain ATCC 19115, strain Jalisco, isolated from a patient in a Hispanic fresh cheese outbreak in 1985, and strain BilMar, originally isolated from a patient in a deli meat outbreak in 1998. The strains were prepared as five-strain cocktails for inoculation. Stock cultures of individual strains were maintained at −70°C in brain heart infusion (BHI; Neogen, Lansing, MI) supplemented with 20% (vol/vol) glycerol. Before use, all bacterial cultures were subjected to two consecutive transfers (24 h at 37°C). Working stocks of each strain were prepared using BHI (Neogen), stored at 4°C, and refreshed on a monthly basis. These working stocks were used to prepare the inoculation cultures.

### MN and SS inoculum preparation.

Macadamia nuts (MN) and sunflower seeds (SS) were purchased from an online vendor (nuts.com, Cranford, NJ). Strains were individually inoculated into 40 ml BHI (Neogen) and incubated for 24 h at 37°C with 200-rpm shaking. Cultures were centrifuged (3,000 × *g*, 15 min, 4°C) and individually resuspended in 5 ml sterile 0.1% buffered peptone water (BPW). The individual BPW strain suspensions (5 ml) were pooled and mixed to obtain a 25-ml suspension of the 5-strain cocktail. The cocktail suspension was recentrifuged (3,000 × *g*, 10 min, 4°C), the supernatant was aspirated, and the remaining pellet was resuspended in 25 ml 0.1% BPW. This liquid pellet was used for MN and SS inoculation. The 25-ml inoculums were mixed with 200 g of macadamia nuts or sunflower seeds. The inoculated nuts or seeds were spread on 2 layers of Whatman paper (GE Healthcare, Chicago, IL), which was on top of 1/4-in mesh screen over a metal tray. Nuts and seeds were allowed to dry for 24 h in a biosafety cabinet. After 24 h, dried nuts and seeds were stored in stomacher bags (Nasco Whirl-Pak, Janesville, WI) for 4 to 6 days and used for heat inactivation trials. The water activities (a_w_s) of ground SS and MN were measured at room temperature using a water activity meter (AquaLab model 3TE; Decagon Devices, Pullman, WA) before inoculation, before inactivation experiments, and after the longest time at each temperature.

### Pecan inoculum preparation.

Strains were individually inoculated into 10 ml BHI (Neogen) and incubated for 24 h at 37°C with 200-rpm shaking. After 24 h, 1 ml of liquid culture was removed from each strain, spread plated on a BHI agar plate (Neogen), and incubated for 24 h at 37°C. Five milliliters of 0.1% BPW was added to each plate, and the plate was gently scraped with a sterile cell scraper (Corning, Corning, NY) to remove inoculum. Twenty-five milliliters of pooled inoculum was collected in a 50-ml conical tube and briefly vortexed. The inoculum was mixed with 200 g of pecans (nuts.com). The inoculated pecans were spread on 2 layers of Whatman paper (GE Healthcare, Chicago, IL), which was on top of 1/4-mesh screen over a metal tray. Pecans were allowed to dry for 24 h in a biosafety cabinet. After 24 h, dried pecans were stored in stomacher bags (Nasco) for 4 to 6 days and used for studies. The water activities (a_w_s) of ground pecans were determined as described above for MN and SS.

### Heat inactivation of macadamia nuts, pecans, and in-shell sunflower seeds.

Heat inactivation was determined by measuring the log CFU reductions between 4 and 80 min at 70°C to 140°C. Fifteen-gram samples of inoculated seeds or nuts were weighed into stomacher bags (Nasco). Three 15-g samples were used to determine the initial counts, and others were used to measure the log reductions at desired time points. The samples were placed on trays made from wire mesh and placed into a hot-air oven (Thermo Fisher, Waltham, MA). The time was measured starting when the dry oven reached the desired temperature. This took approximately 5 min. Following the inactivation time, the seeds or nuts were placed into a new stomacher bag (Nasco) and then immersed in an ice bath for 3 min. To plate the samples, 15 ml 0.1% BPW was added to the seed or nut sample. This was shaken for 1 min. The mixture was rested for 4 min and then shaken for 15 s. The sample was then serially diluted in 0.1% BPW and plated on TSA (Neogen) plates with esculin (Research Products International, Mount Prospect, IL). The plates were incubated for 18 to 24 h at 37°C. Data were analyzed using a log-linear model, and *R*^2^ and *D* values were calculated.

### Heat inactivation of unshelled sunflower seeds.

Heat inactivation was determined by measuring the log CFU reduction between 10 and 30 min at 90°C to 100°C. Fifteen-gram samples of inoculated seeds or nuts were weighed into stomacher bags (Nasco). Three 15-g samples were used to determine initial counts, and the remaining seeds were used to measure the log reductions at desired time points. The samples were placed in a metal strainer (Cuisinart, Stamford, CT) and submerged in an oil bath (Thermo Fisher, Waltham, MA) filled with sunflower seed oil (Great Value, Walmart Stores, Inc., Bentonville, AR). Following the inactivation time, the seeds were placed in a stomacher bag (Nasco) and then immersed in an ice bath for 3 min. To plate the samples, 15 ml 0.1% BPW was added to the seed sample. This was shaken for 1 min. The mixture was rested for 4 min and then shaken for 15 s. The sample was then serially diluted in 0.1% BPW and plated on TSA (Neogen) plates with esculin (RPI Corp.). The plates were incubated for 18 to 24 h at 37°C. The thermal kinetics could not be determined.

### Kinetic parameter calculation and statistical analyses.

After inactivation, the CFU/g data were processed with the log-linear model (33) using Microsoft Excel according to the following equation:

(1) $$N_{t}=N_{0}\cdot e^{\left(-k_{\text{max}}\cdot \;t\right)}$$

where *N_t_* is the population at time *t* (CFU/g), *N*_0_ is the population at time zero (CFU/g), *k*_max_ is the maximum specific inactivation rate (min^−1^), and the *D* value equals ln_10_/*k*_max_. The *Z* value was then calculated according to equation 2, where D* is the first decimal reduction for the temperature T*:

(2) $$\log\;D=\log\;D^{*}\;-\;\left(\frac{T\;-\;T^{*}}{Z}\right)$$

Statistical analyses were performed in R version 4.0.2 (34). A pairwise *t* test was used to test the significance of *D* values between pairs of nut species and temperatures, using individual replicates (*n* = 2) as observations. The confint function in R was used to infer the 95% confidence intervals for the *D* values.

## Supplementary Material

Reviewer comments
