# Supplementary material for: Heat Inactivation of Listeria monocytogenes on Pecans, Macadamia Nuts, and Sunflower Seeds
Source: Microbiol Spectr. 2021 Oct 13;9(2):e01134-21. doi: 10.1128/Spectrum.01134-21 (PMC8515927; doi:10.1128/Spectrum.01134-21)
Supplement: Reviewer comments [file reviewer-comments.pdf]

AMERICAN SOCIETY FOR MICROBIOLOGY | Microbiology Spectrum

Heat Inactivation of *Listeria monocytogenes* on Pecans, Macadamia Nuts, and Sunflower Seeds

Meghan Den Bakker, Henk den Bakker, and Francisco Diez-Gonzalez

Corresponding Author(s): Francisco Diez-Gonzalez, University of Georgia

Review Timeline:

Submission Date: September 7, 2021
Accepted: September 9, 2021

Editor: Jeffrey Gralnick

Reviewer(s): The reviewers have opted to remain anonymous.

Transaction Report:

(Note: With the exception of the correction of typographical or spelling errors that could be a source of ambiguity, letters and reports are not edited. The original formatting of letters and referee reports may not be reflected in this compilation.)

DOI: https://doi.org/10.1128/Spectrum.01134-21

September 9, 2021

Prof. Francisco Diez-Gonzalez
University of Georgia
Center for Food Safety
1109 Experiment St.
Griffin, GA - Georgia 30233

Re: Spectrum01134-21 (Heat Inactivation of *Listeria monocytogenes* on Pecans, Macadamia Nuts, and Sunflower Seeds)

Dear Prof. Francisco Diez-Gonzalez:

Based on your responses and revisions to the prior round of review and in consultation with another Editor, your manuscript has been accepted, and I am forwarding it to the ASM Journals Department for publication. You will be notified when your proofs are ready to be viewed.

The ASM Journals program strives for constant improvement in our submission and publication process. Please tell us how we can improve your experience by taking this quick Author Survey.

As an open-access publication, Spectrum receives no financial support from paid subscriptions and depends on authors' prompt payment of publication fees as soon as their articles are accepted. You will be contacted separately about payment when the proofs are issued; please follow the instructions in that e-mail. Arrangements for payment must be made before your article is published. For a complete list of **Publication Fees**, including supplemental material costs, please visit our website.

Corresponding authors may join or renew ASM membership to obtain discounts on publication fees. Need to upgrade your membership level? Please contact Customer Service at Service@asmusa.org.

Thank you for submitting your paper to Spectrum.

Sincerely,

Jeffrey Gralnick
Editor, Microbiology Spectrum

Journals Department
American Society for Microbiology
1752 N St., NW
Washington, DC 20036
E-mail: spectrum@asmusa.org
